# Complete Genome Sequence of the Siphoviral Bacteriophage Ec-ZZ2, Which Is Capable of Lysing *Enterococcus faecium*

**DOI:** 10.1128/genomeA.01167-16

**Published:** 2016-11-17

**Authors:** Jinghua Li, Hongyan Shi, Chunyan Zhao, Yuchong Hao, Yingze He, Yanbo Sun

**Affiliations:** Department of Pathogen Biology, College of Basic Medical Sciences, Jilin University, Changchun, People’s Republic of China

## Abstract

A virulent bacteriophage (Ec-ZZ2) that infects *Enterococcus faecium* was isolated from sewage. The bacteriophage belongs to the family *Siphoviridae* and has a linear double-stranded DNA genome, with a length of 41,170 bp and a 34.59% G+C content, which is highly similar to *Enterococcus* phage IME-EF4.

## GENOME ANNOUNCEMENT

Enterococci are Gram-positive facultative anaerobic microorganisms that commensally colonize the lower intestinal tract of humans; they are regarded as members of normal microbiota and probiotic agents exerting health benefits on the hosts (1). However, in recent years, *Enterococcus* spp. have emerged as a leading source of nosocomial infections (2, 3), such as bacteremia, endocarditis, and urinary tract infections (4, 5). Furthermore, the development of resistance to vancomycin in *Enterococcus* spp. limits treatment options. One alternative that may overcome the limitation related to antibiotic treatment is phage therapy (6, 7). In this study, a lytic bacteriophage Ec-ZZ2 infecting *Enterococcus faecium* isolates was isolated from a sewage sample obtained from Jilin University First-Affiliated Hospital. The bacteriophage was purified by the agar double-layer method (8, 9) and stored at 4°C. It was classified as a member of the family *Siphoviridae* based on morphological analysis using electron microscopy.

The genomic DNA of Ec-ZZ2 was extracted from purified phage stock using proteinase K-SDS approaches, as described previously (10). The complete genome sequence was determined by Illumina HiSeq 2000 at Shenzhen Huada Gene Technology Service Co., Ltd. (Shenzhen, China). A total of 1,438,298 reads were generated, with 0.49% of low-quality filtered reads. These reads were *de novo* assembled with SOAP*denovo* version 2.04. Gene prediction was carried out using GeneMarkS and further annotated into databases through BLASTp, including KEGG, GO, COG, Swiss-Prot, and IPR.

Phage Ec-ZZ2 contained a linear double-stranded DNA, with a size of 41,170 bp and a G+C content of 34.59%. It has 59 predicted coding sequences (CDSs), five tandem repeats, and two minisatellite DNAs. No tRNA genes were identified. Of the 59 predicted coding sequences, 30 are hypothetical novel or conserved genes, and 29 were given a putative function. This genome contains phage structural genes (major capsid protein, major tail protein, head-tail joining protein, and tail fiber protein), nonstructural genes (DNA polymerase, DNA helicase, DNA primase, and HNH endonuclease). Genes encoding functional proteins related to host lysis include holin and lysin that participate in destroying the peptidoglycan of host cells (11). The analysis of the complete sequence revealed that phage Ec-ZZ2 shows high homology to genomes of the previously reported *Enterococcus* phage IME-EF4 (97% identity, GenBank accession no. KF733017.1) and *Enterococcus* phage EF3 (91% identity, GenBank accession no. KF728385.2), according to the results from the BLASTn program. Furthermore, Ec-ZZ2 contains a putative metallo-beta-lactamase domain protein, which has been described previously (12). Therefore, it will be necessary to clarify the function of this putative drug-resistant gene before Ec-ZZ2 can be used as a therapeutic agent against *Enterococcus* infections.

### Accession number(s).

The whole-genome sequence of bacteriophage Ec-ZZ2 was deposited in GenBank under accession no. KR131750. The version described here is KR131750.1.
